# A Novel DNA Repair‐Gene Model to Predict Responses to Immunotherapy and Prognosis in Patients With EGFR‐Mutant Non‐Small Cell Lung Cancer

**DOI:** 10.1111/1759-7714.70025

**Published:** 2025-02-24

**Authors:** Fen Wang, Xue‐Wu Wei, Ming‐Yi Yang, Chang Lu, Xiao‐Rong Yang, Jia‐Yi Deng, Zhi‐Hong Chen, Qing Zhou

**Affiliations:** ^1^ Guangdong Lung Cancer Institute, Guangdong Provincial People's Hospital (Guangdong Academy of Medical Sciences), Southern Medical University Guangzhou China

**Keywords:** DNA‐damage repair, epidermal growth factor receptor, non‐small cell lung cancer, prognostic model

## Abstract

**Background:**

The epidermal growth factor receptor mutant (EGFRm) non‐small cell lung cancer (NSCLC) has a unique “cold” immune profile. DNA damage repair (DDR) genes are closely related to tumorigenesis and the effectiveness of immunotherapy in many tumors. However, the role and mechanism of DDR in the genesis and progression of EGFRm NSCLC remain unclear.

**Methods:**

This study included 101 EGFRm NSCLC samples from The Cancer Genome Atlas (TCGA) dataset and a GSE31210 dataset (external set) from the GEO database. Cluster analysis was used to identify different subtypes of EGFRm NSCLC based on the expression of DDR genes. Univariate and LASSO regression analysis was used to develop a DDR‐based predictive model. The prognostic significance of this model was assessed using Cox regression, Kaplan–Meier, and receiver operating characteristic (ROC) curve analyses. Bioinformatics analysis was performed to investigate the clinicopathological characteristics and immune profiles associated with this model. In vitro experiment was performed to testify the role of DDR genes in EGFRm NSCLC.

**Results:**

We identified two subtypes of EGFRm NSCLC: DDR‐activated and DDR‐suppressed. The DDR‐activated subtype showed more aggressive clinical behavior and poorer prognosis and was more responsive to immunotherapy. A prognostic model for EGFRm NSCLC was constructed using four DDR genes: *CAPS*, *FAM83A*, *IGLV8‐61*, and *SLC7A5*. The derived risk score could serve as an independent prognostic indicator. High‐ and low‐risk patients exhibited distinct clinicopathological characteristics, immune profiles, and responses to immunotherapy. The T‐cell inflammation and Tumor Immune Dysfunction and Exclusion (TIDE) scores differed between the high‐ and low‐risk subgroups, with both showing enhanced effectiveness of immunotherapy in the low‐risk subgroup. Targeted therapy such as BI.2536, an inhibitor of polo‐like kinase 1, could be effective for patients with high‐risk EGFRm NSCLC. Meanwhile, in vitro detection approved the role of DDR genes in EGFRm NSCLC response.

**Conclusion:**

This study demonstrated a diversity of DDR genes in EGFRm NSCLC and developed a predictive model using these genes. This model could assist in identifying potential candidates for immunotherapy and in assessing personalized treatment and prognosis of patients with EGFRm NSCLC.

## Graphical abstract

We developed a novel DNA damage repair (DDR)‐based prognostic model for EGFR‐mutant non‐small cell lung cancer (NSCLC). Patients were stratified into high risk and low risk subtypes, with significant differences in clinical and biological features. This model highlights a unique molecular approach for predicting prognosis and immunotherapy responses, providing a basis for personalized treatment strategies. (The graph is generated by Figdrow).

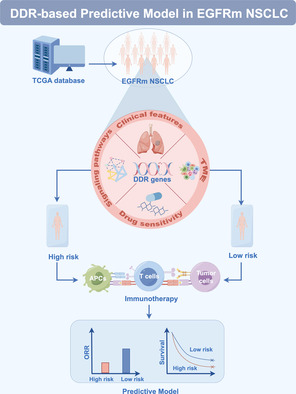

## 1 Introduction

Lung cancer, a common malignancy worldwide, is a primary contributor to cancer‐related mortality [1]. Non‐small cell lung cancer (NSCLC) is the predominant type, comprising 70%–80% of lung cancer [2]. Despite the availability of a range of therapies [3, 4, 5], the prognosis of patients with NSCLC remains poor.

Oncogenic epidermal growth factor receptor (EGFR) plays a key role in the development of NSCLC, especially in Asian populations, where the prevalence of the EGFR mutation is 40%–50% [6]. Both the advanced and early stages of EGFR‐mutant (EGFRm) NSCLC can be effectively treated with EGFR‐tyrosine kinase inhibitors (EGFR‐TKIs) [7]. However, most patients eventually develop resistance to EGFR‐TKIs, and immunotherapy with immune checkpoint inhibitors (ICIs) is a promising strategy for these patients [8, 9]. In solid tumors, the effectiveness of ICIs varies according to the presence of genomic alterations such as deficient‐mismatch repair (dMMR), microsatellite instability‐high (MSI‐H), and high tumor mutation burden (TMB‐H) [10, 11]. The key underlying mechanism behind the variable response to ICIs may be the impaired DNA damage repair (DDR) function.

The DDR signaling pathway plays a vital role in maintaining the genomic stability of human cells. Deficient DDR signaling leads to the accumulation of DNA lesions, which makes oncogenes such as EGFR vulnerable to damage and drives tumorigenesis. Conversely, EGFR activating mutations might be linked to deficiencies in DNA damage repair [12]. EGFR mutations can partly impair DDR by aberrantly regulating the expression of ERCC1 [13]. Tumors with DDR deficiencies accumulate higher genomic instability and neoantigens, which could enhance sensitivity to ICIs [14]. However, EGFRm NSCLC patients typically respond poorly to immunotherapy due to additional factors like low TMB [15, 16], an immunosuppressive tumor microenvironment (TME) [17, 18], and oncogenic signaling pathways [19, 20, 21, 22]. Although DDR impairment might theoretically enhance sensitivity to ICIs, these elements in EGFRm NSCLC counteract the potential benefits of immunotherapy.

To our knowledge, limited research has investigated the genomic and transcriptomic landscape of DDR deficiency in EGFRm NSCLC patients. While EGFRm NSCLC as a whole demonstrates poor sensitivity to ICIs, specific DDR‐related gene signatures may help identify subsets of EGFR‐mutant patients who could derive benefit from immunotherapy. In this study, we explored the heterogeneity of EGFRm NSCLC based on DDR genes using transcriptome data from TCGA. Next, we assessed prognosis, clinical characteristics, and immunophenotypes in patients with different DDR pathways. Based on these analyses, we successfully constructed an independent prognostic model using four DDR genes.

## 2 Methods

### 2.1 Data Acquisition

The raw data of the count matrix and corresponding clinical data of 101 EGFRm NSCLC samples (The samples were approved by the Guangdong Provincial People's Hospital Ethics Committee), including lung squamous cell carcinoma (LUSC) and adenocarcinoma (LUAD), were obtained from The Cancer Genome Atlas (TCGA) database via xenabrowser (https://xenabrowser.net/datapages/). Eligibility required a histological or molecular diagnosis of LUSC or LUAD, with EGFR mutations, and complete transcriptome and clinical data. Exclusion criteria included: tumors without EGFR mutations, non‐LUSC or LUAD, or small cell mixed tumor, incomplete or missing transcriptome and clinical data. The counts of TCGA‐LUAD and TCGA‐LUSC were merged according to the gene name matrix (merging matrix). Moreover, the GSE31210 dataset containing 226 LUAD samples from the Gene Expression Omnibus database (https://www.ncbi.nlm.nih.gov/geo/) was used for external validation.

### 2.2 Cluster Analysis

A total of 84 DDR genes were obtained from GeneGlobe Design & Analysis Hub: RT^2^ Profiler PCR Array Human DNA Damage Signaling Pathway (https://geneglobe.qiagen.com/ro/product‐groups/rt2‐profiler‐pcr‐arrays/PAHS‐029Z). The expression abundance of DDR genes in each sample was evaluated and the EGFRm samples were clustered using the Partitioning Around Medoids (PAM) algorithm with the ConsensusClusterPlus package (Bioconductor version: Development 3.18) [23] based on the expression patterns of DDR genes.

### 2.3 Gene Set Enrichment Analysis

Gene Set Enrichment Analysis (GSEA) was conducted by R statistical software using the Kyoto Encyclopedia of Genes and Genomes (KEGG) and Gene Ontology (GO) database [24, 25].

### 2.4 Identification of Differentially Expressed DDR Genes

The expression levels of DDR genes were transformed into Count Per Million (CPM) with edgeR [26] in R packages. Genes with an average value less than 10 were excluded to construct the final expression matrix, which was fitted into Binomial distribution and tested significance with the Fisher test between subgroups 84 genes retrieved.

### 2.5 Construction of Prognostic Model and Survival Prognosis Analysis

Samples were randomly classified into the training set and the testing set. To investigate the relationship between gene expression levels and survival, a univariate Cox regression analysis was conducted using the survival package on DDR genes from a training set. The prognostic model was constructed by calculating hazard ratios (HR) and *p*‐values for all DDR genes and identifying DDR genes with *p* < 0.05 as potential candidate genes. To further reduce the number of DDR genes, the least absolute shrinkage and selection operator (LASSO) Cox regression analysis was conducted [27]. The time‐dependent receiver operating characteristic (ROC) curves were subsequently used in both of the two sets to evaluate the performance of the DDR prognostic model. The risk score for each sample was predicted using the optimal AUC model, and the samples were then categorized into high‐risk and low‐risk groups based on the median risk score. The risk score is a weighted sum of selected features, with weights given by the LASSO algorithm. This model enhances interpretability by setting less relevant feature coefficients to zero, preventing overfitting. Non‐zero coefficients indicate each feature's contribution to the risk score, allowing for individualized disease risk assessment [28, 29]. Finally, the clinical significance of this risk score was further confirmed through the Kaplan–Meier survival analysis. The correlation analysis between the risk score and the clinical information (including age, sex, and TNM stages) was also carried out.

Based on the above analysis, it is evident that the model has the potential to predict survival prognoses.

### 2.6 Immune Cell Infiltration and Tumor Microenvironment Analysis

To assess the tumor immune microenvironment of EGFRm NSCLC patients, Cibersortx (https://cibersortx.stanford.edu/) was utilized for the analysis of immune cell infiltration [30]. For analyzing the difference in immune cell scores between subgroups, the Wilcox test was applied. T‐cell inflammation score was performed according to the T‐cell inflammation marker genes and their coefficients. T‐cell inflammation scores are often used to predict the prognosis of malignant tumors. The analysis of the disparity in T cell inflammation scores between subgroups was also conducted using the Wilcox Test. In addition, the immune therapy response was analyzed with TIDE (Technology insertion demonstration and evaluation), a computational approach that synthesizes expression signatures of T cell dysfunction and T cell exclusion to model tumor immune evasion [31]. TIDE scores were determined by evaluating cytotoxic T lymphocyte (CTL) levels from a gene expression matrix for each sample. The web application of TIDE is available at http://tide.dfci.harvard.edu. The expression differences between subgroups were compared using the Wilcoxon signed‐rank test.

### 2.7 Drug Sensitive Analysis

We utilized the “pRRophetic” package based on cancer genomics of drug sensitivity data (GDSC, https://www.cancerrxgene.org/) to predict the drug sensitivity between subgroups. Comparisons were made using half of the maximum inhibitory concentration (IC50).

### 2.8 Cell Culture

PC‐9 NSCLC cells were obtained from the Cell Bank of the Shanghai Institute for Biological Sciences, Chinese Academy of Sciences (Shanghai, China). These cells were confirmed to harbor an EGFR mutant genotype. All cells were tested and verified to be free of Mycoplasma. Cells were cultured in RPMI‐1640 supplemented with 1% Penicillin–Streptomycin solution (PBS) and 10% fetal bovine serum (FBS) at 37°C in 5% CO_2_.

### 2.9 Establishment of Overexpression Cell Model

PC‐9 NSCLC cells were seeded in 6‐well plates overnight. NSCLC cells were respectively transfected with *CAPS*, *IGLV8‐6*, *FAM83A*, and *SLC7A5ILT4* overexpression lentivirus by adding 1 mL fresh medium containing lentivirus for 48 h (Genechem Inc). Then the virus transfection enhancer (5 μg/mL) was added to the medium. The medium was replaced using serum‐free medium and cells were cultured for an additional 24 h. Finally, screening PC‐9 cells for stable transfection.

### 2.10 Western Blotting

All cells were harvested and washed twice using phosphate buffered solution and then lysed in leupeptin for half an hour on ice followed by the removal of insoluble material by centrifugation. Equal amounts of protein were loaded into sodium dodecyl sulfate‐polyacrylamide gel electrophoresis (SDS‐PAGE) for electrophoresis and immunoblotted with the indicated antibodies. The primary antibodies were as follows: anti‐Ki‐67 (Abcam, ab15580), anti‐Bcl2 (Abcam, ab182858), and anti‐Caspase (Abcam, ab207802). Subsequently, the membranes were washed and incubated with secondary antibodies. Bands were visualized using the enhanced chemiluminescence (ECL) chemiluminescent detection reagent.

### 2.11 RNA Isolation and Quantitative Real‐Time PCR

All cells were harvested and RNA was extracted using the TRIzol Reagent (TaKaRa) and quantified by the NanoDropND‐1000 Spectrophotometer (Thermo Fisher Scientific). According to the manufacturer's instructions, the cDNA was synthesized from purified total RNA using the HiScript III RT SuperMix for quantitative PCR (Vazyme; Cat No. R323‐01). Quantitative PCR was performed with iTaqTM Universal SYBR Green Supermix (Bio‐Rad). mRNA levels were normalized to β‐Actin mRNAlevels. The specific primers used as follows: KI67: forward, 5′‐ATACGTGAACAGGAGCCAG‐3′, reverse, 5′‐CCTTGGAATCTTGAGCTTTCTC‐3′; BCL2: forward, 5′‐GGATGCCTTTGTGGAACTG‐3′, reverse, 5′‐CAGCCAGGAGAAATCAAACAG‐3′; CASPASE: forward, 5′‐ATCACAGGCATGACAATGC‐3′, reverse, 5′‐TAGTCATGTCCGAAGCAGTG‐3′; β‐actin: forward, 5′‐CTCCATCCTGGCCTCGCTGT‐3′, reverse, 5′‐GCTGCTACCTTCACCGTTCC‐3′.

### 2.12 Transwell In Vitro Cell Migration and Invasion Assays

The lower chamber of the transwell system was added an appropriate amount of serum and 200 uL RPM Imedium was added into the upper chamber. The transwell system was placed at 37°C and incubated in 5% CO_2_ constant temperature incubator for 2 h. Then the cells were digested with 2.5% trypsin and diluted 105 cells to 200 μL RPMI medium without blood serum in the upper chamber. 600 μL RPMI medium with 10% FBS was added in the lower chamber. The transwell system was placed in a constant temperature incubator at 37°C for 48 h. The upper chamber was taken out and wiped with a cotton swab dipped in PBS. Then, cells were fixed with 4% paraformaldehyde and stained with 0.5% crystal violet dye for 15 min, followed by washing with PBS buffer several times. Finally, the cells were observed under a microscope (BX 51, Olympus). Quantitative analysis was performed using Image Pro Plus software (version 6.0; Media Cybernetics, Silver Spring, MD, USA).

### 2.13 Statistical Analysis

Survival curves were generated using the Kaplan–Meier method to assess differences in survival outcomes between the two subgroups. The log‐rank test was employed to determine the statistical significance of the observed differences. To evaluate the potential prognostic value of individual DDR genes, Cox proportional hazards regression analysis was performed. Additionally, both the *t*‐test and the Wilcoxon signed‐rank test were used to compare gene expression levels between the subgroups and assess the significance of the differences. R software (version 4.2.2) was used to perform all statistical analyses and visualizations. In all statistical analyses, significance was determined by *p* < 0.05.

## 3 Results

### 3.1 Classification of Patients With EGFRm NSCLC Based on DDR Genes and Clinical Characteristics Between Subgroups

**FIGURE 1:**
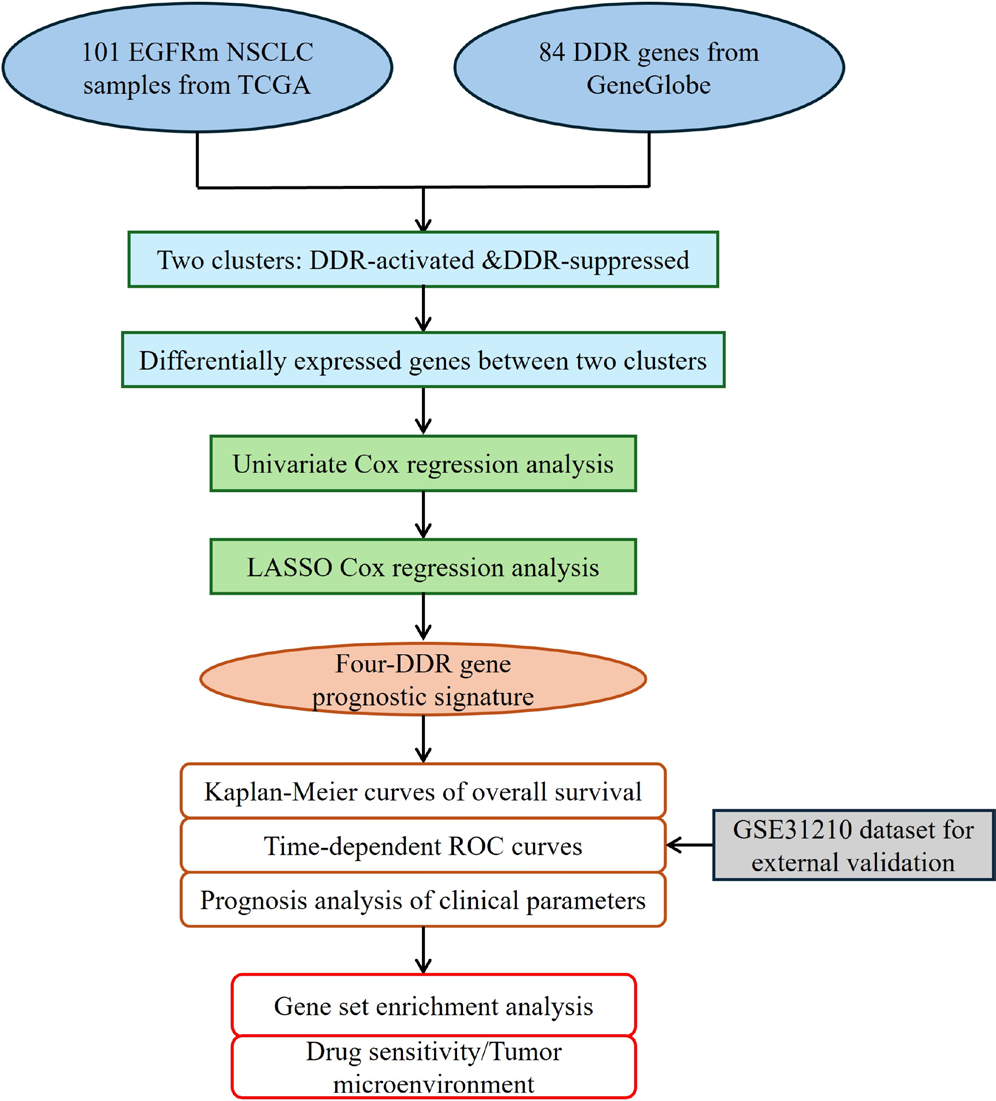
Study flowchart.

**FIGURE 2:**
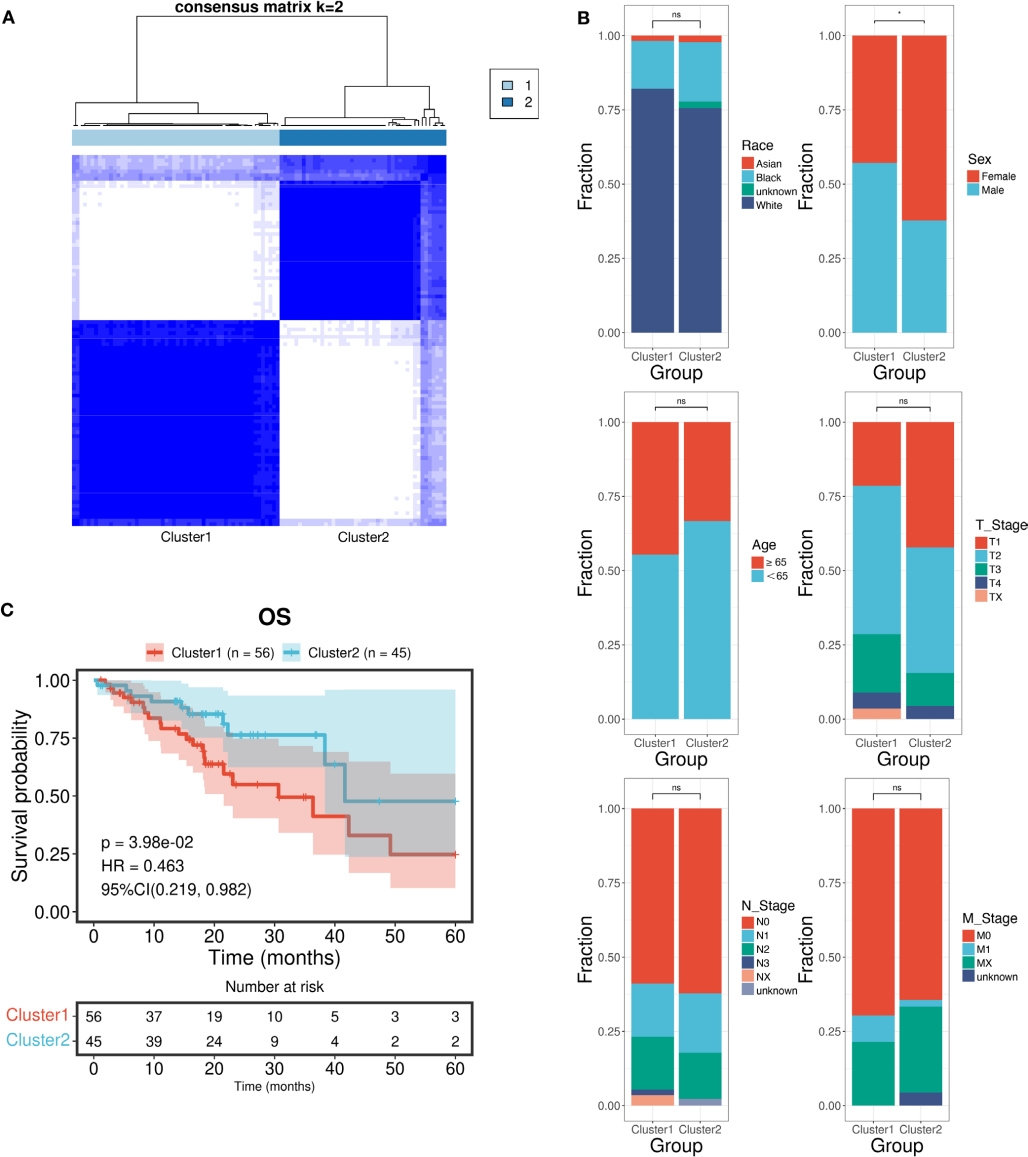
Classification based on differences in DNA damage repair (DDR) genes and clinical characteristics between clusters. (A) Consensus clustering analysis based on DDR gene expression. (B) Evaluation of differences in clinical characteristics between DDR‐activated/suppressed groups. (C) Kaplan–Meier plot showing the overall survival of the two clusters. *p < 0.05, ns, not statistically significant.

Figure 1 shows a flowchart of the study. First, we downloaded transcriptome profiling data and corresponding clinical data of 101 EGFRm NSCLC samples from the TCGA database. The clinical characteristics of these patients are shown in Table 1. We obtained a list of 84 DDR genes (Table S1) and performed consensus clustering analysis to identify different subtypes of EGFRm NSCLC based on the expression pattern of these DDR genes. As shown in Figure 2A, the 101 EGFRm NSCLC patients were divided into two distinct subtypes. Cluster 1 (*n* = 56, 55.4%) was classified as the DDR‐activated subtype because of a notable increase in expression levels of most DDR genes within this cluster. Cluster 2 (*n* = 45, 44.6%) was classified as the DDR‐suppressed subtype because of the comparative decrease in DDR gene expression. We then explored the differences in the clinical features between the subgroups using the t‐test. No significant differences in age, race, or TNM stage were observed between the two subgroups (Figure 2B). Additionally, the DDR‐activated group showed a higher frequency of males (Figure 2B). Furthermore, the survival curves showed that individuals in the DDR‐suppressed subgroup had significantly longer overall survival (OS) than those in the DDR‐activated subgroup (HR, 0.46; 95% confidence interval [CI], 0.219–0.982; *p* = 0.040) (Figure 2C).

**TABLE 1:** The clinical information of 101 EGFRm NSCLC samples.

Characteristic		*N* (%)
Overall		101 (100)
Age, years	63 ± 23	
Race	Asian	2 (1.98)
	Black	18 (17.82)
	White	80 (79.21)
Sex	Female	52 (51.49)
	Male	49 (48.51)
M Stage	M0	68 (67.33)
	M1	6 (5.94)
	MX	25 (24.75)
N Stage	N0	61 (60.40)
	N1	19 (18.81)
	N2	17 (16.83)
	N3	1 (0.99)
	NX	2 (1.98)
T Stage	T1	31 (30.69)
	T2	47 (46.53)
	T3	16 (15.84)
	T4	5 (4.95)
	TX	2 (1.98)

### 3.2 Differential Immune Microenvironment Between the EGFRm NSCLC Subgroups

**FIGURE 3:**
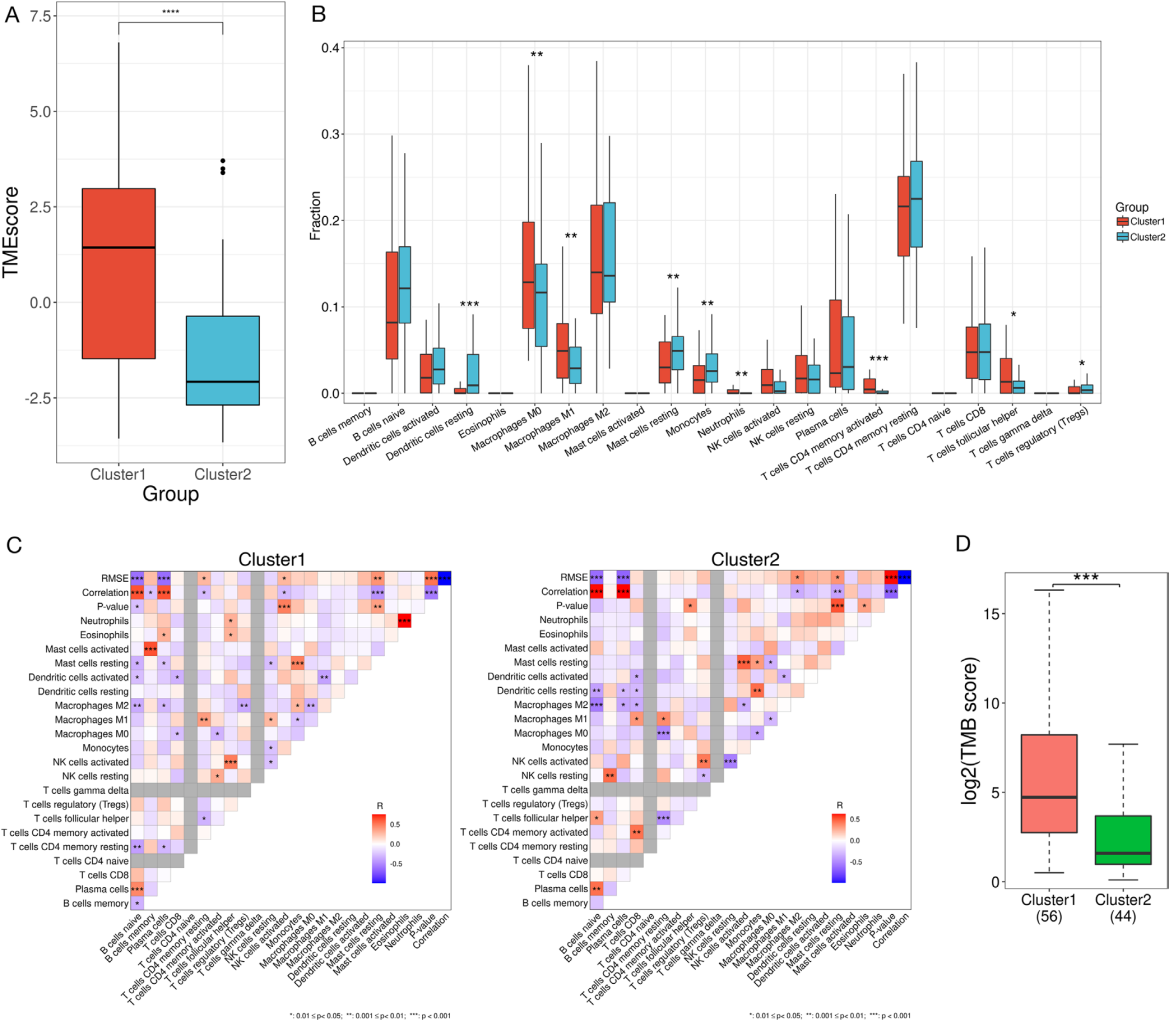
Association between DNA damage repair (DDR) genes and the tumor immune microenvironment (TME). (A) Relationship between the TME score and selected alterations in the DDR genes. (B) Comparison of the immune cell composition between the two clusters. (C) Heatmap showing the correlation between immune cells in the two clusters. (D) Comparison of tumor mutation burden (TMB) in the two clusters. *p < 0.05, **p < 0.01, ***p < 0.001.

The TME refers to the internal environment in which tumors originate and reside. Previous studies have revealed the role of the TME in cancer progression and its response to therapy [32]. Increasing evidence has shown that the TME is crucial to the outcomes of immunotherapy [33]. We compared the TME score using the Wilcoxon signed‐rank test. to reveal the immunotherapeutic responses between the two EGFRm NSCLC subtypes. The TME score of the DDR‐activated subtype was significantly higher than that of the DDR‐suppressed subtype (Figure 3A).

The TME comprises both tumor and nontumor cells including immune cells, fibroblasts, and endothelial cells, and the activity of these cells, particularly immune cells, is closely linked to tumor growth [34, 35]. Thus, we analyzed the immune cell fraction in the different subpopulations utilizing the Wilcoxon signed‐rank test. The results showed that the proportions of M0 (*p* < 0.01) and M1 macrophages (*p* < 0.01), neutrophils (*p* < 0.01), CD4 memory‐activated T cells (*p* < 0.001), and follicular helper T cells (*p* < 0.05) were significantly higher in the DDR‐activated subgroup than those in the DDR‐suppressed subgroup (Figure 3B). In contrast, we observed higher fractions of resting dendritic cells (*p* < 0.001), resting mast cells (*p* < 0.01), monocytes (*p* < 0.01), and regulatory T cells (Tregs) (*p* < 0.05) in the DDR‐suppressed subgroup (Figure 3B).

To investigate the causes of the variations in the immune microenvironment observed in the two subgroups, we conducted an additional analysis of the relationship between immune cells. The correlation heatmap showed that the interaction patterns of immune cells differed between the two subgroups. In the DDR‐activated subgroup, there was a positive interaction between activated natural killer (NK) cells and follicular helper T cells (*p* < 0.001), whereas, in the DDR‐suppressed subgroup, there was a positive interaction between the activated NK cells and the Tregs (*p* < 0.01). Tregs were negatively correlated with M2 macrophages in the DDR‐activated subgroup (*p* < 0.01). The neutrophil and eosinophil counts were strongly positively correlated in the DDR‐activated subgroup (*p* < 0.001), whereas this correlation was absent in the DDR‐suppressed subgroup (Figure 3C).

The accumulation of uncorrected DNA damage is closely linked to genetic traits such as high TMB, resulting in DDR modifications [36]. Thus, the differences in gene mutations between the two subgroups based on TMB were also analyzed utilizing the Wilcoxon signed‐rank test. The TMB score was higher in the DDR‐activated subgroup than in the DDR‐suppressed subgroup (Figure 3D).

### 3.3 Construction and Validation of DDR‐Related Gene Prognostic Signature

**FIGURE 4:**
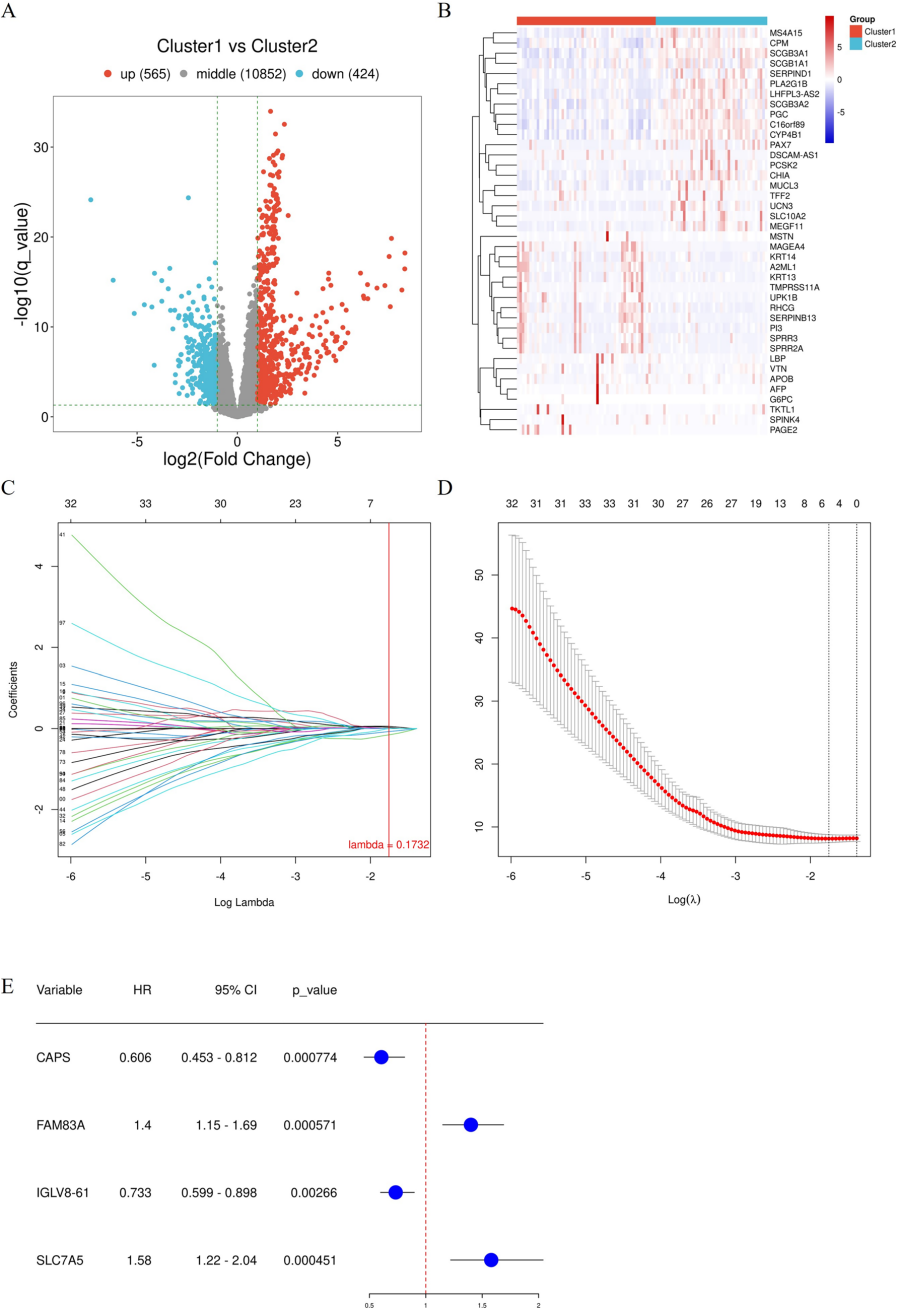
Identification of hub DDR genes associated with the prognosis. (A) Volcano plot showing differentially expressed genes (DEGs) between the two clusters. (B) Heatmap showing the top 20 DEGs in the two clusters. (C) Coefficient profiles of the LASSO regression model. (D) Cross‐validation for tuning parameter screening in the LASSO regression model. (E) Results of LASSO‐Cox regression analysis of hub DDR genes.

Considering the importance of DDR pathways in predicting lung cancer prognosis, we developed a DDR‐based predictive model for patients with EGFRm NSCLC. Differentially expressed genes (DEGs) between the two subtypes of EGFRm NSCLC were identified. Overall, compared with the DDR‐suppressed subtype, there were 565 upregulated genes and 424 downregulated genes in the DDR‐activated subtype (Figure 4A, Table S2). The top 20 DEGs are shown in a heatmap (Figure 4B). The profiling landscape of mRNA variation revealed the different mutational landscapes between the subgroups. To develop the DDR gene‐based prognostic model, we performed univariate Cox regression analysis on all 989 DEGs and identified 109 genes associated with the prognosis. The samples were randomly divided into a training set and a testing set with a ratio of 1:1. To prevent overfitting, LASSO‐Cox regression analysis was further performed on the training set (Figure 4C,D) and four genes were selected as hub DDR genes, including *CAPS*, *FAM83A*, *IGLV8‐61*, and *SLC7A5* (Figure 4E). The HR of *CAPS* and *IGLV8‐61* was < 1, whereas the HR of FAM83A and SLC7A5 was > 1.

**FIGURE 5:**
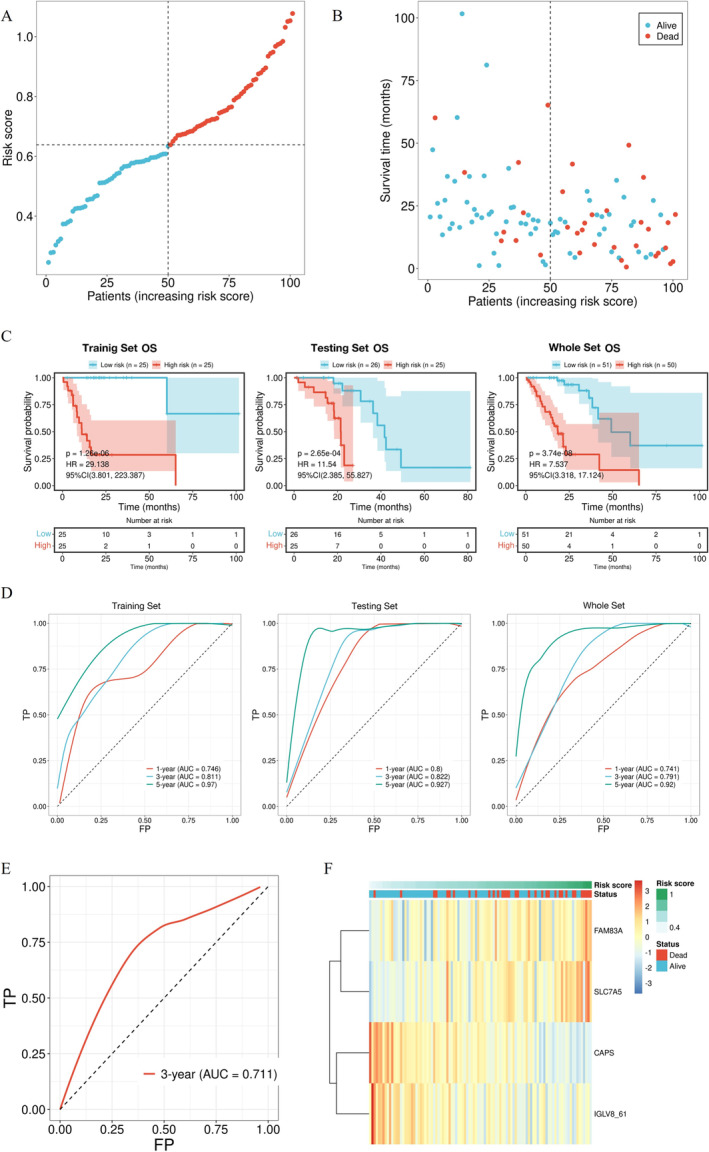
Construction and validation of the prognostic model. (A) Risk score plot. (B) The relationship between risk scores and survival status of each patient. (C) Kaplan–Meier survival curves for high‐risk and low‐risk patients in the training set, testing set, and whole set. (D) ROC curves of the prognostic model in the training set, testing set, and whole set. (E) ROC curve of the prognostic model in the external set. (F) Clustering heatmap of hub DDR genes.

The regression coefficient of these four genes and their expression levels were combined to calculate the risk score for each patient (Figure 5A). We divided the patients into high‐risk and low‐risk groups using the median risk score. Higher risk scores were correlated with shorter survival time of the patients (Figure 5B). Based on the median value of the risk score, the patients were divided into high‐risk and low‐risk groups. Survival curves were plotted using the Kaplan–Meier method to evaluate the prognostic value of the model for the OS in EGFRm NSCLC. The high‐risk group had a significantly lower OS than the low‐risk group in the training set, testing set, and whole set (Figure 5C). ROC curves were used to further assess the prognostic accuracy of the model. The 3‐year AUCs for the training set, testing set, and whole set were 0.811, 0.822, and 0.791, respectively (Figure 5D). Moreover, an external set (GSE31210) was also used for the validation. As shown in Figure 5E, the AUC value of the ROC curve for the 3‐year OS in the external set was 0.711. Additionally, the clustering heatmap demonstrated that patients with higher risk scores of *FAM83A* and *SLC7A5* exhibited higher risk score status and worse survival, whereas *CAPS* and *IGLV8‐61* showed opposite results (Figure 5F).

### 3.4 Analysis of the Correlation Between Risk Scores and Clinical Characteristics of EGFRm NSCLC Patients

**FIGURE 6:**
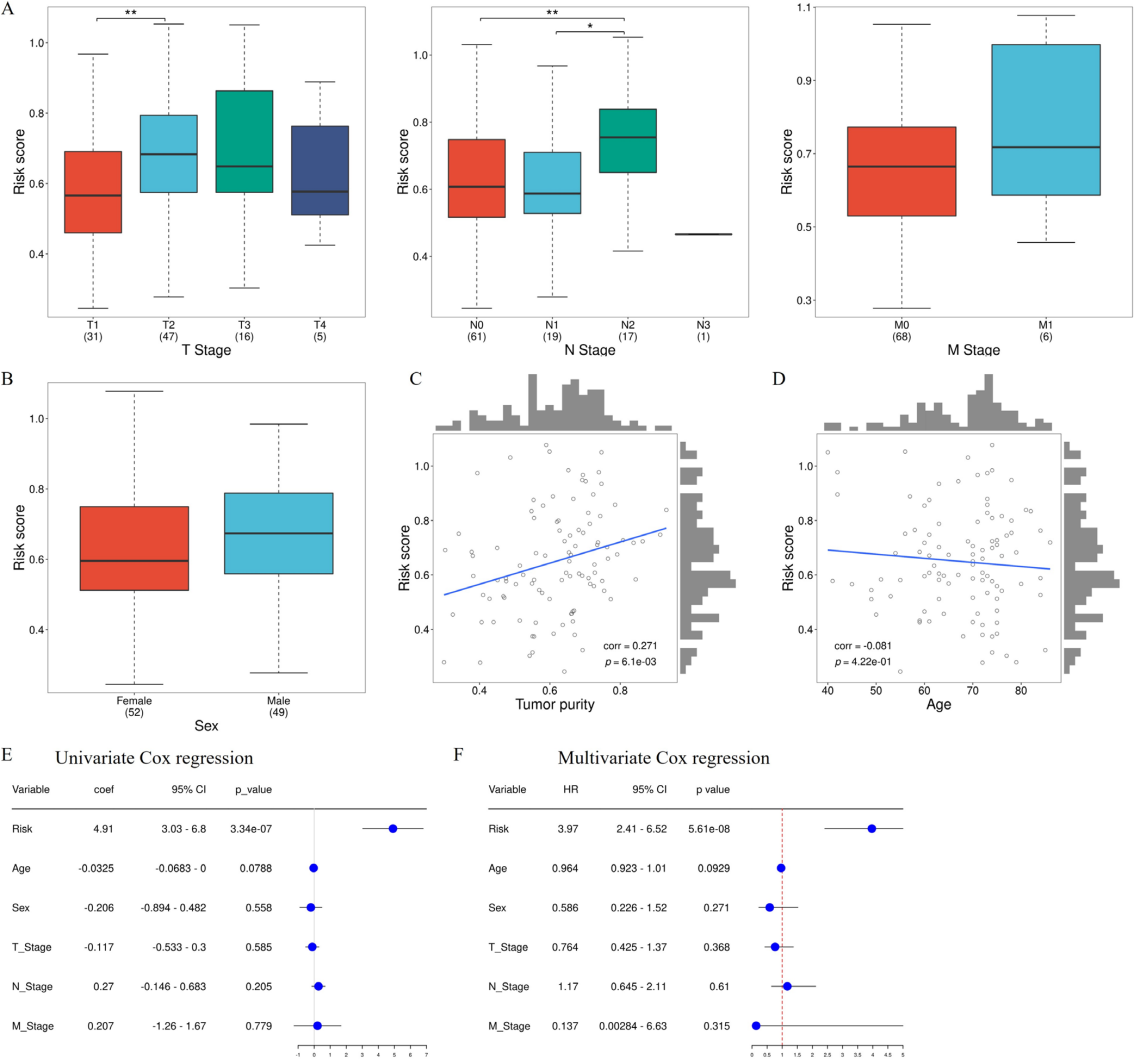
Analysis of correlations between clinical features and risk scores. (A) Difference in risk scores based on TNM stages. (B) Difference in risk scores based on sex. (C) Correlation between risk scores and tumor purity. (D) Correlation between risk scores and age. (E) Univariate cox regression analysis for prognosis. (F) Multivariable cox regression analysis for prognosis. **p < 0.01.

We then evaluated the correlation between clinical features and risk scores using the *t*‐test. The risk scores were compared in various TNM staging subgroups (Figure 6A). The risk scores were significantly higher in the T2 subgroup than in the T1 subgroup (*p* < 0.01) and in the N2 subgroup than in the N0 and N1 subgroups (*p* < 0.05 or 0.01). However, no significant difference was observed between the M0 and M1 subgroups, as well as other T or N subgroups. The risk scores also did not significantly differ between different sexes (Figure 6B). Figure 6C illustrated a statistically significant positive correlation between tumor purity, defined as the proportion of tumor cells within the tumor tissue, and the calculated risk scores. Additionally, although not significant (*p* = 0.422), higher risk scores were negatively correlated with patient age (Figure 6D). Furthermore, both univariate and multivariable Cox regression analysis demonstrated that the risk score was an independent prognostic factor for patients with EGFRm NSCLC (Figure 6E,F).

**FIGURE 7:**
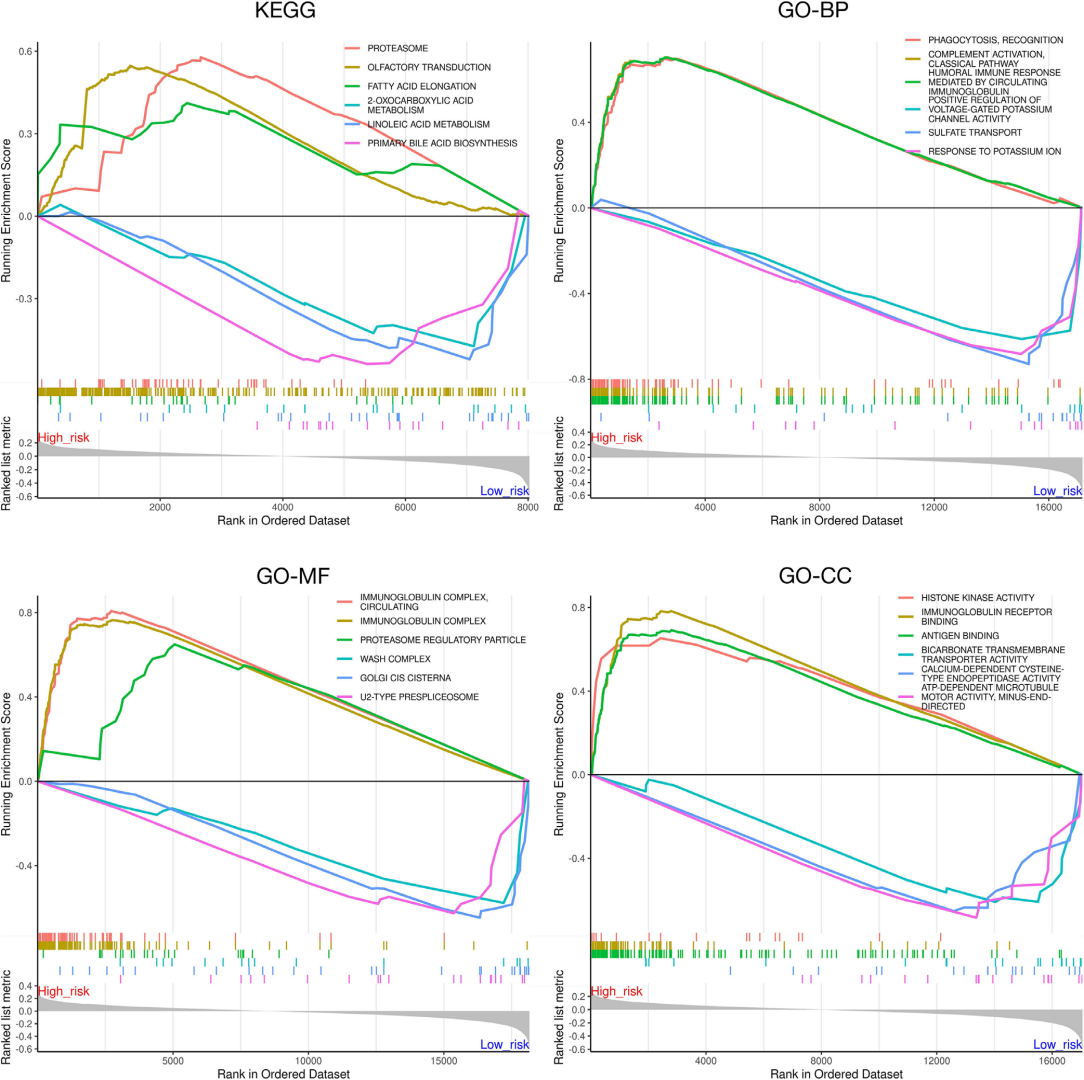
GSEA of specific pathways enriched by the high‐risk and low‐risk groups.

We conducted a gene set enrichment analysis (GSEA) to identify specific signaling pathways associated with the risk score. In general, the high‐risk group was enriched in the humoral immunity‐related pathways, whereas the low‐risk group was enriched in the potassium regulation‐related pathways (Figure 7).

### 3.5 Drug Sensitivity Analysis and Evaluation of Effectiveness of Immunotherapy

**FIGURE 8:**
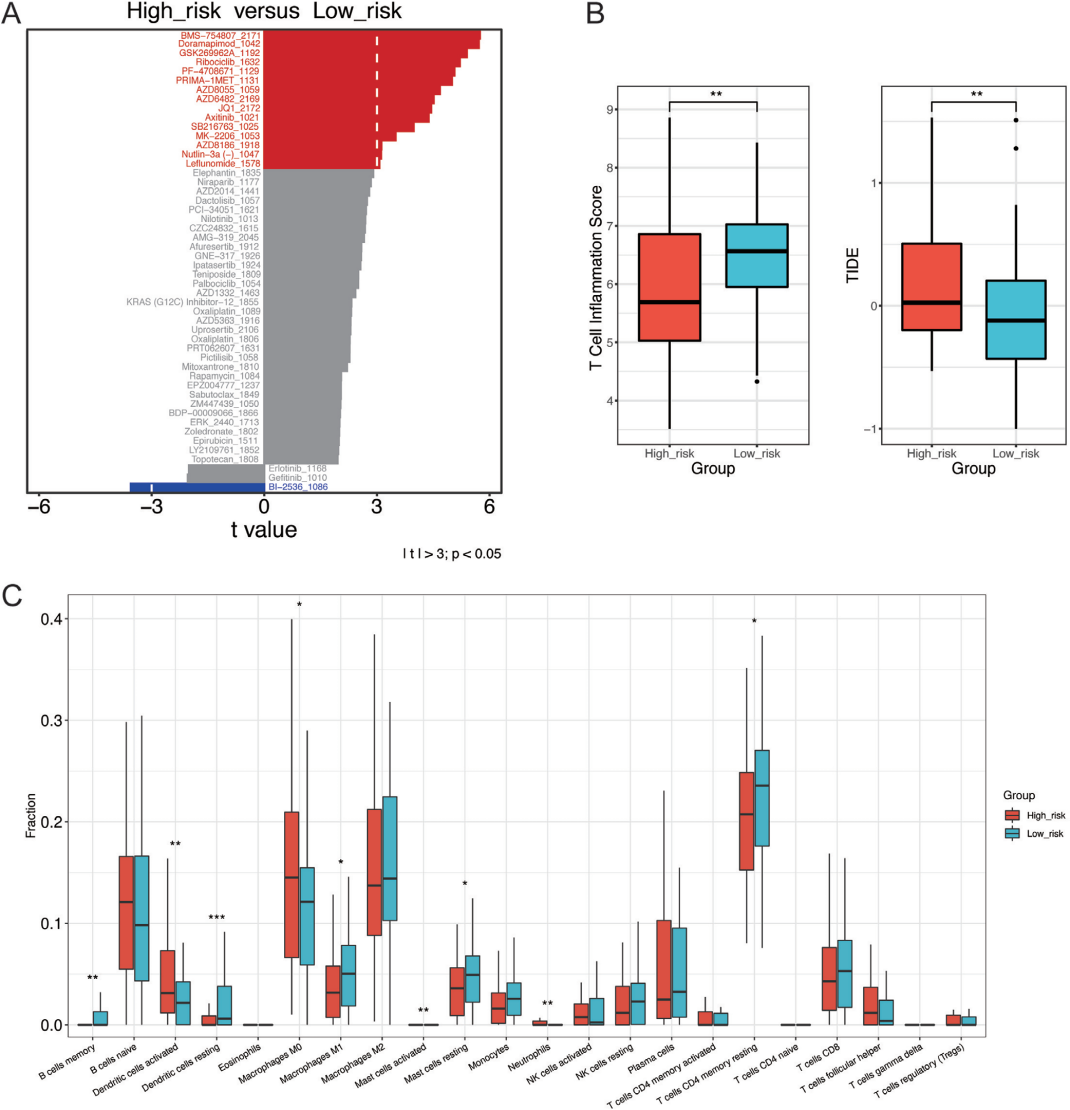
Drug sensitivity and tumor immune microenvironment (TME) analysis of the prognostic model. (A) Drug sensitivity analysis. (B) Comparison of the T‐cell inflammation score (left) and Tumor Immune Dysfunction and Exclusion (TIDE) score (right) in the low‐ and high‐risk groups. (C) Comparison of the immune cell composition in the low‐ and high‐risk groups. *p < 0.05, **p < 0.01, ***p < 0.001.

We evaluated the differences in the sensitivity of lung cancer therapeutic drugs between the low‐risk and high‐risk groups using IC50 values. The top 50 drugs that differed most significantly between the two groups are listed in Figure 8A. We noted that BI.2536 (polo‐like kinase 1 inhibitor) had a considerably lower IC50 in patients in the high‐risk group compared with those in the low‐risk group, indicating that BI.2536 may be effective for treating individuals classified as high‐risk.

To further verify the applicability of our prognostic model, we analyzed the T‐cell inflammation and TIDE scores utilizing the Wilcoxon signed‐rank test. The high‐risk subgroup had a lower T‐cell inflammation score (*p* < 0.01) and a higher TIDE score (*p* < 0.01), whereas the low‐risk group had the opposite pattern (Figure 8B). The TME analysis revealed that the low‐risk subgroup exhibited significantly higher levels of B memory cells (*p* < 0.01) and resting dendritic cells (*p* < 0.001). Conversely, the high‐risk group exhibited significantly higher levels of activated dendritic cells (*p* < 0.01) and neutrophils (*p* < 0.01) (Figure 8C).

### 3.6 The Influence of DDR‐Related Gene on NSCLC Cells In Vitro

**FIGURE 9:**
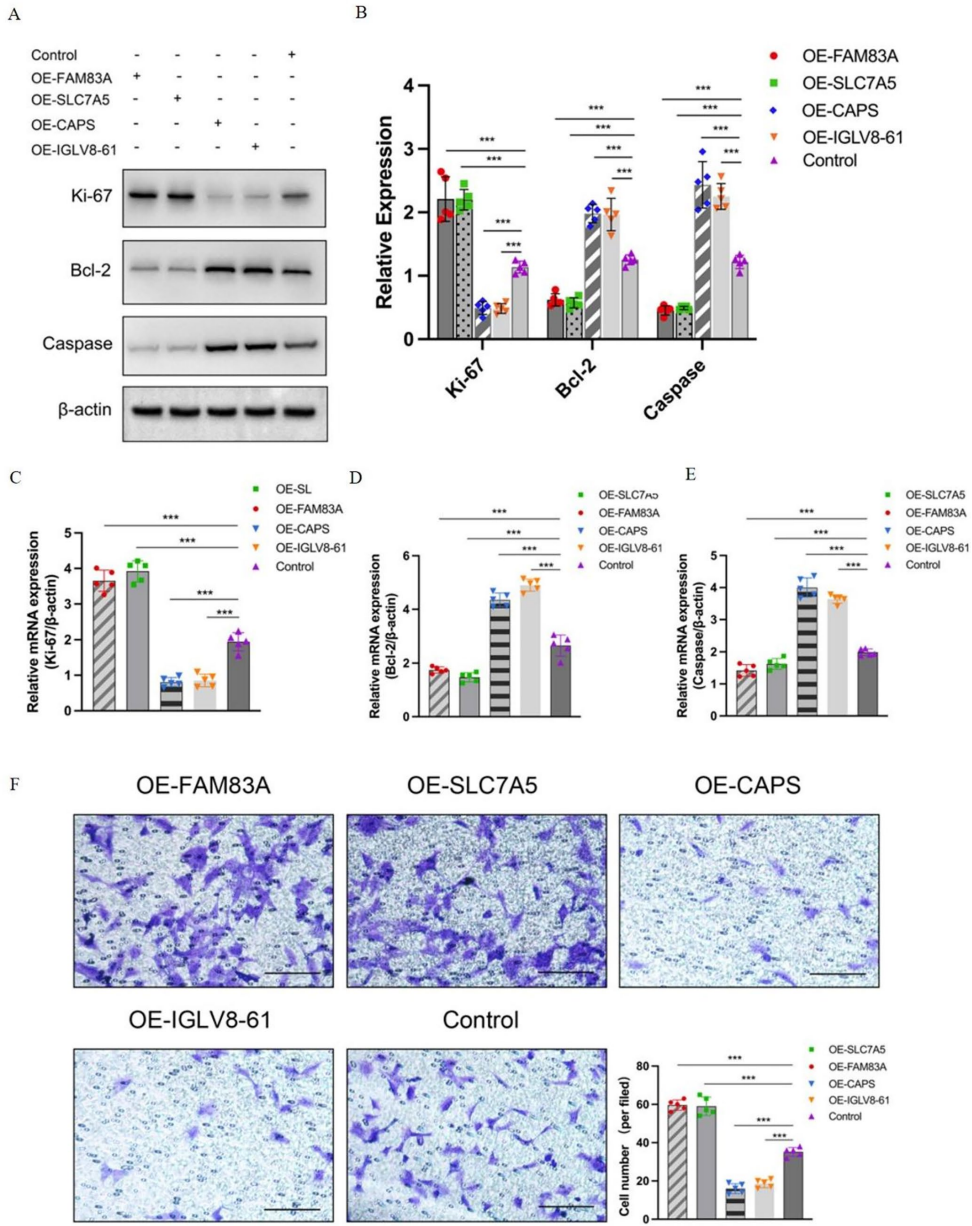
The influence of DDR‐related gene on NSCLC cells in vitro. (A) The protein content in five groups with western blotting analysis. (B) The quantitative analysis results. (C) Then the quantitative real‐time PCR assay results showed that the expression trend of Ki‐67 in each group. (D) Then the quantitative real‐time PCR assay results showed that the expression trend of Bcl‐2 in each group. (E) Then the quantitative real‐time PCR assay results showed that the expression trend of caspase in each group. (F) The transwell assay showed that compared with the control group, more cells could be found in the OE‐FAM83A group and OE‐SLC7A5 group, with lower cell number in the OE‐CAPS group and OE‐IGLV8‐61 group. *p < 0.05, **p < 0.01, ***p < 0.001. Bar = 200 μm.

NSCLC cells were cultured according to the guidelines. Figure 9A showed that the expression level of Ki‐67 was higher in the OE‐FAM83A group and OE‐SLC7A5 group compared with the control group. On the contrary, in comparison with the control group, lower expression of ki‐67 was observed in the OE‐CAPS group and OE‐IGLV8‐61 group. As for the expression of Bcl‐2 and capsease, the OE‐CAPS group and OE‐IGLV8‐61 group showed lower expression levels compared to the control group. Conversely, compared to the control group, the higher expression of Bcl‐2 and caspase was shown in the OE‐FAM83A group and OE‐SLC7A5 group. The results of quantitative analysis with differences also proved the above trend as shown in Figure 9B (*p* < 0.01). Then the quantitative real‐time PCR assay was performed and the results showed that the expression trend of Ki‐67, Bcl‐2, and caspase in each group was consistent with that of WB detection (Figure 9C–E) (*p* < 0.01). The transwell assay showed that compared with the control group, more cells could be found in the OE‐FAM83A group and OE‐SLC7A5 group, with lower cell number in the OE‐CAPS group and OE‐IGLV8‐61 group (Figure 9F) (*p* < 0.01).

## 4 Discussion

EGFR‐TKI resistance is inevitable in advanced EGFRm lung cancer. Of patients with failure of first‐line osimertinib treatment regimens, the resistance mechanism is unknown in approximately 70% [37], necessitating the use of later‐line targeted therapy. Immunotherapy combined with bevacizumab has been shown to improve survival in patients with EGFRm lung cancer after EGFR‐TKI failure but is associated with a high incidence of adverse events [8, 9]. Hence, novel biomarkers are required for predicting the prognosis and effectiveness of immunotherapy in patients with EGFRm NSCLC.

Various DRR genes have been found to be involved in the development of cancer. These genes have been used as markers for evaluating the prognosis of many types of cancer, including colon cancer [38], lung adenocarcinoma [39], and prostate cancer [40]. DDR dysfunction can enlarge somatic mutations and lead to an accumulation of intracellular DNA fragments, resulting in increased neoantigen accumulation and enhanced immunogenicity [41]. DDR gene alterations have putative predictive value not only in chemotherapy but also in immunotherapy in patients with cancer [42, 43, 44]. Moreover, DDR gene alterations are positively correlated with TMB [45, 46, 47], making it a promising biomarker for predicting response to ICIs.

EGFR is vital for DNA repair, as it translocate to the nucleus to engage with DNA repair proteins and manage DDR after EGFR‐TKIs, radiotherapy and chemotherapy [48, 49, 50, 51, 52]. EGFRm NSCLC exhibit impaired repair of double‐strand breaks via the non‐homologous end joining pathway [53, 54, 55, 56]. The progression of the cell cycle induced by EGFR activation may impede the cell‐cycle arrest required for efficient DNA repair processes [57]. However, several well‐documented mechanisms attribute the resistance of EGFRm NSCLC to immunotherapy. These tumors tend to exhibit lower TMB compared to other NSCLC subtypes, thereby limiting neoantigen production and subsequent immune recognition [15, 16]. EGFR‐mutant tumors are often characterized by reduced CD8+ T cell infiltration, lower PD‐L1 expression, and higher infiltration of immunosuppressive cells (e.g., regulatory T cells, M2 macrophages) [17, 18]. EGFR‐driven oncogenic pathways themselves can suppress immune activation through mechanisms such as reduced MHC class I expression and secretion of immunosuppressive cytokines [19, 20, 21, 22]. Thus, while DDR impairment may theoretically increase tumor sensitivity to ICIs, these additional factors in EGFRm NSCLC create an immunosuppressive milieu that negates the expected benefits of immunotherapy.

Emerging evidence from a few recent studies suggest that the efficacy of ICIs is relatively more favorable in a specific group of EGFRm NSCLC patients [58, 59, 60, 61, 62]. Patients harboring EGFR L858R mutation have shown more benefit from immunotherapy than those with EGFR exon 19 deletion (ORR, 7% in EGFR 19 deletion subgroup versus 16% in L858R subgroup). The preferential response of NSCLC patients with uncommon EGFR mutations to PD‐1 blockade therapy has also been observed in clinical practice [63, 64, 65]. A recent study demonstrated that neoadjuvant immunochemotherapy in early‐stage lung cancer with EGFR mutations resulted in a major pathological remission rate of 44%, significantly surpassing the efficacy observed in historical controls treated with EGFR‐TKIs [66]. Thus, not all EGFRm NSCLC patients are resistant to immunotherapy. Screening for specific subgroups may facilitate the personalized application of ICIs in patients with EGFRm NSCLC.

Given the intrinsic relationship between EGFR mutations and DDR, and the crucial function of DDR genes in the progression of lung cancer, w334e conducted a bioinformatics analysis to assess the predictive and prognostic significance of the associated DDR genes in patients with EGFRm NSCLC. Our model aims to dissect the heterogeneity within EGFR‐mutant tumors by leveraging DDR‐related gene signatures, potentially uncovering a subgroup with a more favorable immune profile.

In this study, we found that the expression of DDR genes can affect the clinical outcomes of EGFRm NSCLC patients and further identified two distinct DDR statuses in this population: DDR‐activated and DDR‐suppressed subgroups. The status of DDR was not related to the clinicopathological stage of the tumor, but differed significantly according to sex. There were more male patients in the DDR‐activated subgroup than in the DDR‐suppressed subgroup, which may be because male patients were more likely to be smokers and nitrosamine in tobacco induces genomic instability [67]. Although patients in the DDR‐activated subgroup had a poorer prognosis than those in the DDR‐suppressed subgroup, they had more frequent gene mutations, higher TMB and TME scores, and a more inflammatory composition of immune cells, indicating that NSCLC patients with some DDR gene subtypes may still respond to immunotherapy even if they harbor EGFR‐sensitive mutations.

Next, we identified a novel prognostic model for patients with EGFRm NSCLC using four DDR genes (*CAPS*, *FAM83A*, *IGLV8‐61*, and *SLC7A5*). The genes in the model are strongly associated with both carcinogenesis and antitumor therapy [68]. Calcyphosin (CAPS) is a protein that is encoded by the *CAPS* gene. It binds to calcium through an EF‐hand and is involved in the cAMP and calcium‐phosphatidylinositol pathways [69, 70]. *CAPS* is recognized as a possible prognostic indicator in various human carcinomas, including lung cancer [71, 72, 73]. In gliomas [74], the interaction between *CAPS* and *MYPT1*, a crucial controller of protein phosphatase 1C, has been discovered, highlighting its significant role in cell cycle regulation. While CAPS lacks direct evidence of involvement in DDR, its role in calcium homeostasis might indirectly affect DDR pathways, especially during cellular stress responses. The immunoglobulin variable lambda 8–61 (*IGLV8‐61*) gene, which encodes a variable region of the immunoglobulin light chain lambda, is part of the adaptive immune system and plays a role in antigen recognition according to Gene Bank data. *IGLV8‐61* contributes to B‐cell receptor diversity via somatic hypermutation (SHM) and class‐switch recombination (CSR), which involve intentional DNA breaks and repairs through non‐homologous end joining (NHEJ) and mismatch repair (MMR) [75]. Research indicates that dysregulation or mutation of *IGLV* genes, including *IGLV8‐61*, may contribute to tumor progression or immune evasion, but their exact role remains uncertain and requires more study [76, 77]. In our study, *CAPS* and *IGLV8‐61* displayed HR less than 1 in the LASSO‐Cox regression analysis, suggesting a potential protective role in EGFRm NSCLC development. Consistently, these genes exhibited the ability to inhibit the proliferation, apoptosis, and metastasis of EGFRm NSCLC cell in vitro.

*FAM83A* is the smallest of the Family with sequence similarity 83 (FAM83) members. A recent study suggests that *FAM83A* could be an important cancer biomarker because of its conserved DUF1669 N‐terminal domain [78]. Several types of cancers have been shown to have an overexpression of the FAM83A oncogene, which has been linked to a negative prognosis [79, 80]. In NSCLC, *FAM83A* amplification promotes tumorigenicity via the ERK and PI3K/Akt/mTOR pathways [81] and serves as a prognostic predictor [79, 82]. Its overexpression in cancer cells may disrupt DDR mechanisms, leading to genomic instability and increased resistance to DNA‐damaging treatments like radiation and chemotherapy [83]. Solute carrier family 7 member 5 (*SLC7A5*), also known as L‐type amino acid transporter 1 (*LAT1*), is responsible for the transport of carboxylic acids, thyroid hormones, and xenobiotics. The encoding protein LAT1 exchanges glutamine for neutral amino acids, including essential amino acids that are required for cancer cell proliferation and growth. The expression of *SLC7A5* has been studied in various cancers, and an elevated expression is associated with a poor prognosis [84]. *SLC7A5* regulates mTOR signaling, which plays a role in cellular stress responses like DNA damage [85]. Enhanced metabolism and nutrient uptake can indirectly affect DNA damage repair (DDR) by supplying essential repair resources. In cancer, SLC7A5 dysregulation may disrupt DDR and lead to therapy resistance. In our study, these two genes were linked to poor survival outcomes in EGFRm NSCLC, aligning with previous research. Additionally, cell line experiments demonstrated their role in promoting tumor growth and metastasis. The four DDR genes are essential to the development of tumors and contribute to treatment resistance, so they may be useful as both biomarkers and therapeutic targets. In addition to gene expression, alternative allelic variants also play a significant role in influencing tumor biology. Our investigation of the dbSNP and dbVar databases identified the presence of copy number variants (CNVs) and single nucleotide variants (SNVs) in the *FAM83A* and *SLC7A5* genes. Furthermore, SNVs were detected in the *CAPS* gene, while CNVs were observed in the *IGLV8‐61* gene.

Meanwhile, detailed investigations into the molecular pathways and interactions between DDR genes and the immune system have been performed in recent years. Before the recognition of adaptive immunity, the innate immune system plays a crucial part in recruiting immune cells to tumors [53]. Recent studies have shown that the STING pathway plays an important role in the host's innate immune system against cancer, driving the production of interferon (IFN) and triggering T cell responses [54, 55, 56]. Some research also indicated that DDR deficiencies could activate the innate immune system by cyclic GMP–AMP synthase‐stimulator of interferon genes pathway. As for the adaptive immune system, DDR gene deficiencies could improve the tumor detection of this procedure to withstand the tumor progress. On the other hand, excessive DDR gene expression could arouse immune responses by cell death signals. Overall, elucidating the pathways between DDR genes and the immune system could offer deeper insights into their roles in tumor immunity and resistance mechanisms. Further research is needed to explore these relationships in more detail.

In this study, the risk score derived from the model was significantly correlated with the OS in patients with EGFRm NSCLC regardless of the clinicopathological features. The high‐ and low‐risk subgroups had notable disparities in their T‐cell inflammation and TIDE scores, suggesting that the low‐risk subgroup is more likely to respond to immunotherapy. The T‐cell inflammation score was positively associated with the response to immunotherapy, whereas higher scores were associated with an increased likelihood that the tumor would escape immune surveillance. The TIDE score, which represents tumor immune dysfunction and rejection, is frequently used to evaluate the potential for immune escape based on the gene expression pattern in tumor specimens. A higher TIDE score indicates an enhanced immune escape capacity of the tumor, resulting in poorer ICIs response. Moreover, the two subgroups exhibited distinct immune cell compositions, also suggesting that the low‐risk subgroup is more likely to respond to immunotherapy. This implies that our prediction model could be used not only to predict OS, but also to predict the response to immunotherapy in patients with EGFRm NSCLC. In order to elucidate underlying associations between specific phenotypes and molecular functions or pathways, we conducted the GSEA analysis. High‐risk subgroup was enriched in humoral immunity‐related pathways, suggesting its potential links to immune dysregulation, chronic inflammation, or potential immune evasion. In contrast, low‐risk subgroup showed enrichment in potassium regulation pathways, indicating better maintenance of ionic homeostasis, reduced migration potential, and less aggressive behavior [86, 87]. These findings elucidate distinct characteristics between high‐and low‐risk subgroups, potentially offering a novel molecular foundation for risk stratification. Further validation in experimental studies would be essential to confirm these findings.

The treatment of EGFRm‐related cancer has centered on the key factor, EGFR. A dysfunctional epithelial‐mesenchymal transition (EMT) is one of the mechanisms of tumor cell dissemination that leads to metastasis and poor outcomes [57]. The studies approved that PRMT5 could orchestrate EGFR and AKT networks to promote EMT in the tumor cells. In our report, some treatment methods and targets have been systematically evaluated. One potential drug (BI2536) for patients with high‐risk EGFRm NSCLC was screened based on our constructed model. The model predicted that BI2536 was more likely to be effective in high‐risk patients than in low‐risk patients. BI2536 targets polo‐like kinase 1 (PLK1) and has potential for the treatment of NSCLC. In vivo experiments have confirmed that BI2536 not only induces pyroptosis through the caspase‐3/GSDME pathway but also increases CD8^+^ T‐cell infiltration in tumor sites [88, 89]. Moreover, inhibition of PLK1 by BI2536 could reduce gefitinib‐induced hepatotoxicity by restoring COX6A1 expression without impairing the anti‐cancer activity of gefitinib [90]. In an open‐label, randomized phase II clinical trial, BI2536 monotherapy showed modest efficacy and favorable safety in patients with relapsed NSCLC [91]. These findings support the further development of PLK1 inhibitors for treating patients with high‐risk EGFRm NSCLC.

Indeed, this study has some limitations that should be acknowledged. First, the sample size was limited due to relying on data from the GEO and TCGA public databases. Additionally, limitations in the data prevented us from performing a hierarchical analysis according to the type of EGFR mutation. And the potential biases in dataset selection, such as observer bias, exclusion bias, and measurement deviation should be overcome. While we did include the GSE31210 dataset for external validation, we recognize that this single dataset may not fully represent the diversity of EGFRm NSCLC patients across different populations and clinical settings. Prospective cohort studies in clinical settings are warranted in future. Second, the absence of in vivo validation hinders our comprehension of the role of four genes identified in NSCLC progression and immunotherapy response. Additional animal studies and clinical validation are needed to clarify their biological importance and potential for translation. Furthermore, it remains uncertain whether this model demonstrates superiority over established biomarkers, such as PD‐L1 and TMB. Last, the challenges of translating bioinformatics findings into clinical practice need to be valued, potentially leading to more personalized and effective treatment approaches.

Nevertheless, our study reveals a new model for the prognosis of DDR genes in EGFRm NSCLC. This model, acting as an independent predictor for OS in both the training and testing sets, accurately predicted the prognosis of patients diagnosed with EGFRm NSCLC. To the best of our knowledge, this is the first study to comprehensively explore the heterogeneity of DDR genes in EGFRm NSCLC and to integrate these findings into a novel prognostic model. Moreover, our study provides novel insights into the interaction between DDR pathways, immune phenotypes, and potential therapeutic targets, which could guide personalized treatment strategies for EGFRm NSCLC patients. The four genes we examined have not yet been documented in the context of their relevance to immunotherapy. Therapies targeting the four DDR genes or PLK1 should be investigated as potential combination therapies.

## 5 Conclusions

In summary, this study demonstrated the diversity of DDR in EGFRm NSCLC and developed a unique predictive model based on DDR genes. This model could assist with identifying potential candidates for immunotherapy and in providing personalized treatment and prognosis for patients with EGFRm NSCLC.

## Author Contributions

**Fen Wang:** conceptualization, methodology, software, investigation, formal analysis, writing – original draft. **Xue‐Wu Wei:** data curation, writing – original draft. **Ming‐Yi Yang:** visualization, investigation. **Chang Lu:** resources, supervision. **Xiao‐Rong Yang:** software, validation. **Jia‐Yi Deng:** visualization, writing – review and editing. **Zhi‐Hong Chen:** resources, supervision; **Qing Zhou:** conceptualization, funding acquisition, resources, supervision, writing – review and editing. All authors read and approved the final manuscript.

## Ethics Statement

All experiments were approved by Research Ethics Committee of Guangdong Provincial People's Hospital (No. GDRECKY2020‐199‐01).

## Conflicts of Interest

Qing Zhou reports honoraria from AstraZeneca, Boehringer Ingelheim, BMS, Eli Lilly, MSD, Pfizer, Roche, and Sanofi, outside the submitted work.

## Supporting information


**DATA S1.** Supporting Information.

## Data Availability

The datasets used and analyzed during the current study are available from the corresponding author on reasonable request.
