# Supplementary material for: A Novel DNA Repair‐Gene Model to Predict Responses to Immunotherapy and Prognosis in Patients With EGFR‐Mutant Non‐Small Cell Lung Cancer
Source: Thorac Cancer. 2025 Feb 24;16(4):e70025. doi: 10.1111/1759-7714.70025 (PMC11850292; doi:10.1111/1759-7714.70025)
Supplement: Supplementary file 1 — DATA S1. Supporting Information. [file TCA-16-e70025-s001.docx]

**Supplementary Appendix**

Supplementary Table 1. DNA damage repair (DDR) genes list.

| Νο | Symbol | GeneBank | Description |
| --- | --- | --- | --- |
| 1 | ABL1 | NM_005157 | C-abl oncogene 1, non-receptor tyrosine kinase |
| 2 | APEX1 | NM_080649 | APEX nuclease (multifunctional DNA repair enzyme) 1 |
| 3 | ATM | NM_000051 | Ataxia telangiectasia mutated |
| 4 | ATR | NM_001184 | Ataxia telangiectasia and Rad3 related |
| 5 | ATRIP | NM_032166 | ATR interacting protein |
| 6 | ATRX | NM_000489 | Alpha thalassemia/mental retardation syndrome X-linked |
| 7 | BARD1 | NM_000465 | BRCA1 associated RING domain 1 |
| 8 | BAX | NM_004324 | BCL2-associated X protein |
| 9 | BBC3 | NM_014417 | BCL2 binding component 3 |
| 10 | BLM | NM_000057 | Bloom syndrome, RecQ helicase-like |
| 11 | BRCA1 | NM_007294 | Breast cancer 1, early onset |
| 12 | BRIP1 | NM_032043 | BRCA1 interacting protein C-terminal helicase 1 |
| 13 | CDC25A | NM_001789 | Cell division cycle 25 homolog A (S. pombe) |
| 14 | CDC25C | NM_001790 | Cell division cycle 25 homolog C (S. pombe) |
| 15 | CDK7 | NM_001799 | Cyclin-dependent kinase 7 |
| 16 | CDKN1A | NM_000389 | Cyclin-dependent kinase inhibitor 1A (p21, Cip1) |
| 17 | CHEK1 | NM_001274 | CHK1 checkpoint homolog (S. pombe) |
| 18 | CHEK2 | NM_007194 | CHK2 checkpoint homolog (S. pombe) |
| 19 | CIB1 | NM_006384 | Calcium and integrin binding 1 (calmyrin) |
| 20 | CRY1 | NM_004075 | Cryptochrome 1 (photolyase-like) |
| 21 | CSNK2A2 | NM_001896 | Casein kinase 2, alpha prime polypeptide |
| 22 | DDB1 | NM_001923 | Damage-specific DNA binding protein 1, 127kDa |
| 23 | DDB2 | NM_000107 | Damage-specific DNA binding protein 2, 48kDa |
| 24 | DDIT3 | NM_004083 | DNA-damage-inducible transcript 3 |
| 25 | ERCC1 | NM_001983 | Excision repair cross-complementing rodent repair deficiency, complementation group 1 (includes overlapping antisense sequence) |
| 26 | ERCC2 | NM_000400 | Excision repair cross-complementing rodent repair  deficiency, complementation group 2 |
| 27 | EXO1 | NM_130398 | Exonuclease 1 |
| 28 | FANCA | NM_000135 | Fanconi anemia, complementation group A |
| 29 | FANCD2 | NM_033084 | Fanconi anemia, complementation group D2 |
| 30 | FANCG | NM_004629 | Fanconi anemia, complementation group G |
| 31 | FEN1 | NM_004111 | Flap structure-specific endonuclease 1 |
| 32 | GADD45A | NM_001924 | Growth arrest and DNA-damage-inducible, alpha |
| 33 | GADD45G | NM_006705 | Growth arrest and DNA-damage-inducible, gamma |
| 34 | H2AFX | NM_002105 | H2A histone family, member X |
| 35 | HUS1 | NM_004507 | HUS1 checkpoint homolog (S. pombe) |
| 36 | LIG1 | NM_000234 | Ligase I, DNA, ATP-dependent |
| 37 | MAPK12 | NM_002969 | Mitogen-activated protein kinase 12 |
| 38 | MBD4 | NM_003925 | Methyl-CpG binding domain protein 4 |
| 39 | MCPH1 | NM_024596 | Microcephalin 1 |
| 40 | MDC1 | NM_014641 | Mediator of DNA-damage checkpoint 1 |
| 41 | MLH1 | NM_000249 | MutL homolog 1, colon cancer, nonpolyposis type 2 (E. coli) |
| 42 | MLH3 | NM_014381 | MutL homolog 3 (E. coli) |
| 43 | MPG | NM_002434 | N-methylpurine-DNA glycosylase |
| 44 | MRE11A | NM_005590 | MRE11 meiotic recombination 11 homolog A (S. cerevisiae) |
| 45 | MSH2 | NM_000251 | MutS homolog 2, colon cancer, nonpolyposis type 1 (E. coli) |
| 46 | MSH3 | NM_002439 | MutS homolog 3 (E. coli) |
| 47 | NBN | NM_002485 | Nibrin |
| 48 | NTHL1 | NM_002528 | Nth endonuclease III-like 1 (E. coli) |
| 49 | OGG1 | NM_002542 | 8-oxoguanine DNA glycosylase |
| 50 | PARP1 | NM_001618 | Poly (ADP-ribose) polymerase 1 |
| 51 | PCNA | NM_182649 | Proliferating cell nuclear antigen |
| 52 | PMS1 | NM_000534 | PMS1 postmeiotic segregation increased 1 (S. cerevisiae) |
| 53 | PMS2 | NM_000535 | PMS2 postmeiotic segregation increased 2 (S. cerevisiae) |
| 54 | PNKP | NM_007254 | Polynucleotide kinase 3'-phosphatase |
| 55 | PPM1D | NM_003620 | Protein phosphatase, Mg2+/Mn2+ dependent, 1D |
| 56 | PPP1R15A | NM_014330 | Protein phosphatase 1, regulatory (inhibitor) subunit 15A |
| 57 | PRKDC | NM_006904 | Protein kinase, DNA-activated, catalytic polypeptide |
| 58 | RAD1 | NM_002853 | RAD1 homolog (S. pombe) |
| 59 | RAD17 | NM_002873 | RAD17 homolog (S. pombe) |
| 60 | RAD18 | NM_020165 | RAD18 homolog (S. cerevisiae) |
| 61 | RAD21 | NM_006265 | RAD21 homolog (S. pombe) |
| 62 | RAD50 | NM_005732 | RAD50 homolog (S. cerevisiae) |
| 63 | RAD51 | NM_002875 | RAD51 homolog (S. cerevisiae) |
| 64 | RAD51B | NM_133509 | RAD51 homolog B (S. cerevisiae) |
| 65 | RAD9A | NM_004584 | RAD9 homolog A (S. pombe) |
| 66 | RBBP8 | NM_002894 | Retinoblastoma binding protein 8 |
| 67 | REV1 | NM_016316 | REV1 homolog (S. cerevisiae) |
| 68 | RNF168 | NM_152617 | Ring finger protein 168 |
| 69 | RNF8 | NM_183078 | Ring finger protein 8 |
| 70 | RPA1 | NM_002945 | Replication protein A1, 70kDa |
| 71 | SIRT1 | NM_012238 | Sirtuin 1 |
| 72 | SMC1A | NM_006306 | Structural maintenance of chromosomes 1A |
| 73 | SUMO1 | NM_003352 | SMT3 suppressor of mif two 3 homolog 1 (S. cerevisiae) |
| 74 | TOPBP1 | NM_007027 | Topoisomerase (DNA) II binding protein 1 |
| 75 | TP53 | NM_000546 | Tumor protein p53 |
| 76 | TP53BP1 | NM_005657 | Tumor protein p53 binding protein 1 |
| 77 | TP73 | NM_005427 | Tumor protein p73 |
| 78 | UNG | NM_003362 | Uracil-DNA glycosylase |
| 79 | XPA | NM_000380 | Xeroderma pigmentosum, complementation group A |
| 80 | XPC | NM_004628 | Xeroderma pigmentosum, complementation group C |
| 81 | XRCC1 | NM_006297 | X-ray repair complementing defective repair in Chinese hamster cells 1 |
| 82 | XRCC2 | NM_005431 | X-ray repair complementing defective repair in Chinese hamster cells 2 |
| 83 | XRCC3 | NM_005432 | X-ray repair complementing defective repair in Chinese hamster cells 3 |
| 84 | XRCC6 | NM_001469 | X-ray repair complementing defective repair in Chinese hamster cells 6 |

Supplementary Table 2. Overview of DEGs in two clusters.

| Data information | | Difference threshold | | No. of DEGs | | Samples | |
| --- | --- | --- | --- | --- | --- | --- | --- |
| Data source | Platform | FC | Q value | Up-regulated | Down-regulated | Cluster1 | Cluster2 |
| LUSC+LUAD | Sequencing | 2 | 0.05 | 424 | 565 | 56 | 45 |

DEG: differentially expressed genes.
